# Time series modelling and forecasting of mpox incidence and mortality in Nigeria

**DOI:** 10.1186/s12879-025-11174-0

**Published:** 2025-06-04

**Authors:** Emmanuel Afolabi Bakare, Oluwaseun Akinlo Mogbojuri, Dolapo Oluwaseun Oniyelu, Afeez Abidemi, Deborah Oluwatobi Daniel, Idowu Isaac Olasupo, Samuel Abidemi Osikoya, Aaron Onyebuchi Nwana, Ronke Dorcas Olorunfemi, Samson Oluwafemi Olagbami

**Affiliations:** 1 International Centre for Applied Mathematical Modelling and Data Analytics, Federal University Oye-Ekiti, Ekiti State, Nigeria; 2 Department of Mathematics, Federal University Oye-Ekiti, Ekiti State, Nigeria; 3 Department of Mathematical Sciences, Adekunle Ajasin University, Akungba-Akoko, Nigeria; 4 Department of Computer Science, Federal University Oye-Ekiti, Ekiti State, Nigeria; 5 Department of Mathematical Sciences, Federal University of Technology, Akure, Nigeria; 6 Department of Animal and Environmental Biology, Federal University Oye-Ekiti, Ekiti State, Nigeria

**Keywords:** Mpox, Time series analysis, Wavelet analysis, ARIMA, Forecasting

## Abstract

The World Health Organization (WHO) declared mpox a Public Health Emergency of International Concern (PHEIC) twice, in response to the global outbreak, first in May 2022 and again in August 2024, after a span of 2 years and 3 months. African countries continue to be a hotspot for the ongoing mpox outbreaks and Nigeria has contributed substantially in exporting the virus to other countries, highlighting the need for an in-depth analysis of outbreak patterns and forecasting to inform public health policy. This study used the Auto-Regressive Integrated Moving Average (ARIMA) model to perform a 14-month forecast of mpox cases and mortality in Nigeria using mpox monthly routine data. The data were split into two portions; 70% for training, used to estimate the parameters of the forecasting model and 30% for testing, used to evaluate the model’s accuracy. Wavelet analysis was used to decompose the time series into its various frequency components, enabling a multi-resolution analysis of the data. The ARIMA model forecasted an average of 13 mpox cases per month and zero mortality over a 14-month period. The wavelet power spectrum revealed a strong annual cycle between June 2022 and June 2023. In order to sustain the forecasted downward trend in mpox cases in the coming months, it is essential that the National Mpox Technical Working Group (TWG) of Nigeria Centre for Disease Control and Prevention (NCDC) continue to coordinate scale up of vaccine coverage and improve surveillance especially in high risk area. The findings will ultimately improve focused interventions and knowledge of mpox outbreak patterns by guiding public health policy, allocating resources optimally, and preparing health systems for potential outbreaks.

## Introduction

Mpox (previously called monkeypox) has attracted attention in recent times due to the 2022 global outbreaks, especially in regions with little or no previously documented cases and the global resurgence in 2024 [1, 2]. Mpox is a zoonotic disease caused by the Mpox virus (MPXV), a species of the genus *Orthopoxvirus* within the *Poxviridae* family of viruses [1, 3]. Two major, distinct genetic clades of the virus have been identified by phylogenetic evidence: the Congo Basin clade, also known as Clade I and the West African clade, commonly called Clade II; with the former being more virulent [1, 3, 4]. While Clade I, with subclades Ia and Ib is endemic to Central Africa; Clade II, with subclades IIa and IIb is endemic to West Africa [4, 5]. Historically, the first documented case of human mpox was from a 9-months-old child in 1970 in the Democratic Republic of Congo (DRC), followed by six cases among children in Sierra Leone, Liberia, and Nigeria between 1970 and 1971 [2, 6]. The mpox virus can be transmitted to humans through contact with infected humans or animals, or contact with objects that are contaminated with the virus [1, 7]. There are also possibilities of human to animal transmission as well as vertical transmission during pregnancy or birth [1, 8, 9].

The World Health Organization (WHO), prompted by the sudden global upsurge, declared mpox a Public Health Emergency of International Concern (PHEIC) twice, first in May 2022 and then again over two years later, in August 2024 [1]. The current global outbreak, which started in early 2022 has recorded a total of 124,753 laboratory confirmed cases including 272 mortalities in 128 countries as of 31 December 2024 [10]. The three most affected countries between January 2022 and December 2024 are the United States of America (34,490 cases), the Democratic Republic of the Congo (15,340 cases), and Brazil (13,429 cases) with these three countries accounting for about 50% of the total global cases [10]. In 2024, African countries reported over 20,000 mpox cases, including more than 600 confirmed and suspected deaths, attributed to both Clade I and Clade II [11].

Nigeria experienced an mpox outbreak in September 2017, nearly 40 years after the previous confirmed cases, making it the largest documented outbreak of the West African clade before the 2022 global outbreak [12]. As of 19 January 2025, Nigeria has recorded a total of 1,056 laboratory confirmed mpox cases including 9 confirmed deaths (case fatality ratio of 0.9%) being the fourth most affected country in Africa after Democratic Republic of Congo, Burundi and Uganda [10]. Most of the mpox cases in Nigeria in recent times were recorded among adults aged 25 years and older, primarily among sexually active males, with the high incidence partly linked to an increase in male-to-male sexual relationships among Africans [6].

Several control measures such as limited contact with rodents, isolation of patients etc have been put in place by the Nigeria Centre for Disease Control and Prevention (NCDC) to prevent the spread of mpox disease in Nigeria [12]. Currently, there is no specific treatment of mpox, however, antivirals such as tecovirimat and brincindofovir have been recommended for treatment of mpox in humans [4, 12]. In August 2024, Nigeria received 10,000 doses of the Jynneos vaccine becoming the first African country to receive mpox vaccines [13]. The mpox vaccination campaign in Nigeria which was initiated in November 2024, targeted healthcare workers and individuals with weakened immune systems in hospitals across the Federal Capital Territory (FCT), Abuja [13].

In recent years, forecasting models have become increasingly important, especially for newly emerging disease outbreaks, playing a crucial role in addressing public health challenges and managing these outbreaks effectively [14]. Several studies have employed time series modelling to understand the dynamics of infectious diseases including mpox outbreaks. In [14], the authors applied the auto-regressive integrated moving average (ARIMA) to study outbreak trends of mpox in the ten hardest hit countries in the world. Using data spanning the period from May 18, 2022, to December 31, 2022, they provided a short-term forecast up to January 20, 2023. Their results show that the USA is most likely to be the worst-hit country, with an average of 58 cases per day (95% CI: 0–400).

The authors in [15] used time series modelling methods such as ARIMA and joinpoint regression to forecast mpox trends in the four most affected countries in Africa including Nigeria. The study utilised data between August 6, 2023, to August 18, 2024 from the four countries with their forecasting models indicating a sustained rise in mpox cases. A time series ensemble technique was proposed by [16]. The proposed method was then used to obtain efficient short-term mpox disease forecasts in the USA, Brazil, France, Spain. By employing an ensemble sub-epidemic modelling technique, the authors in [17] generated short-term forecasts of new mpox cases across seven countries. The study made use of data spanning from the week of July 28, 2022, to the week of October 13, 2022, to produce 4-week ahead forecasts of laboratory-confirmed cases for the study areas. Various other studies, including [18–22], have also applied machine learning algorithms and time series techniques to forecast the trends of infectious diseases across the globe.

Wavelet analysis has emerged as a useful technique for identifying hidden patterns of epidemiological time series and providing valuable insights into the underlying mechanisms of epidemiological processes [23, 24]. Various studies have been carried out in recent decades using wavelet analysis to analyse times-series of infectious disease [25], starting with the work done by [23] where wavelet analysis was used to reveal synchrony patterns of measles in the UK. By applying the wavelet approach, the authors in [26] showed that there exists a strong but non-stationary correlation between dengue outbreaks, El Niño dynamics, and precipitation in Thailand. The epidemiological features of influenza virus and its correlation with climatic factors in Jinan, China were investigated by the authors in [27] using wavelet analysis. The study shows that influenza dynamics were defined by an annual cycle, with notable winter epidemic peaks occurring between December and February of the period under consideration. Wavelet analysis was also used to detect the seasonal trends of influenza in Zhejiang province, China from 2009 to 2022 by the authors in [28]. In more recent times, several research studies such as [29–32] have also applied wavelet analysis to COVID-19 pandemic.

Despite the numerous forecasting studies conducted to understand global mpox trends, there is a paucity of research specifically focused on forecasting mpox outbreaks in Nigeria. This is particularly worrisome given Nigeria’s significant role in exporting the mpox virus to other countries, especially as the Clade IIb virus responsible for the 2022 global mpox outbreak originated from Nigeria [1, 33, 34]. Thus, understanding the trends of mpox outbreaks in Nigeria is crucial for curbing its spread and controlling the transmission of the virus, not only in Africa but globally [33]. This study therefore aims to bridge this gap in knowledge by identifying seasonal trends and hidden patterns in the outbreak of mpox in Nigeria, and forecasting the trends and burden of mpox in Nigeria over the next two years. This is done by applying the Auto-Regressive Integrated Moving Average (ARIMA) model in combination with wavelet analysis. The remaining part of the paper is organised as follows: The materials and methods employed in this research are discussed in “Materials and methods” section. In “Results and discussions” section, the results and discussions are presented. Finally, the concluding remarks are given in “Conclusion” section.

## Materials and methods

### Study setting

**Fig. 1:**
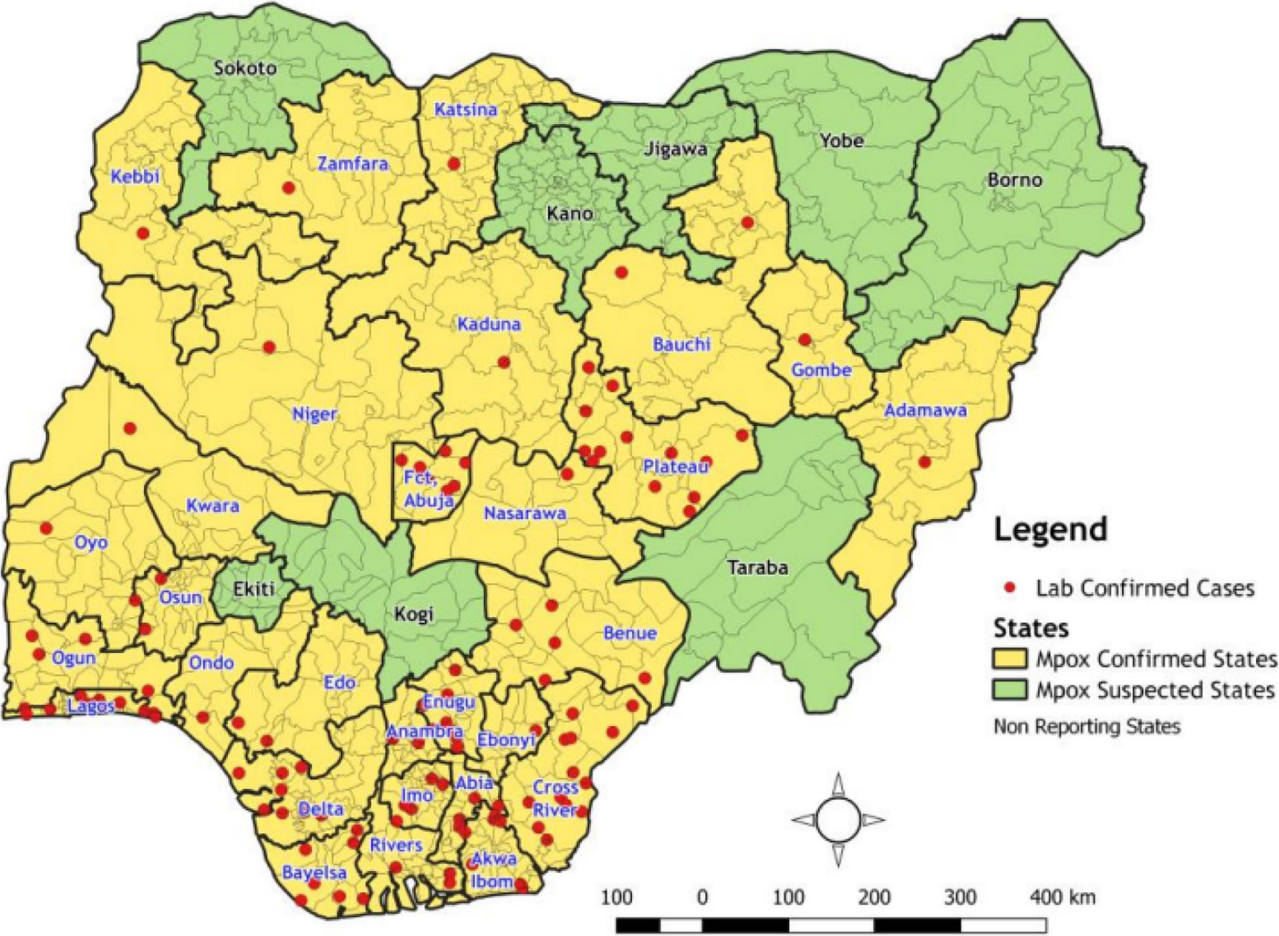
Map of Nigeria showing suspected and confirmed mpox cases reported across 36 states and the FCT, with confirmed cases in 28 states and the FCT (Source: [36])

The study was conducted using aggregated data from all the states in Nigeria. Nigeria is located in West Africa and has a latitude of 9.0820$^\circ$N and a longitude of 8.6753$^\circ$E. It shares boundary with the Republic of Benin, Niger, Chad and Cameroun. It has 36 states and the Federal Capital Territory (FCT), Abuja. Nigeria’s estimated population was 211,493,324 in 2021 and 216,783,381 in 2022 [35]. Figure 1 presents the map of Nigeria, showing the geographic distribution of suspected and confirmed cases of mpox in all 36 states including the FCT, Abuja. The map shows that the highest number of cases were reported from states in the South-South region of Nigeria.

### Data description

The dataset used in this study comprises monthly reported cases of mpox and related mortality in Nigeria, spanning a period of 46 months - from January 2021 to October 2024. The aggregated data from different states in Nigeria were obtained from NCDC. The dataset includes 46 observations, each representing a month, and three main variables which include information on number of suspected mpox cases, laboratory-confirmed cases, and mortality.

### ARIMA model

The ARIMA model is a commonly used statistical method for analysing and forecasting time series data [14, 15]. It is based on the assumption of stationarity, that is, the mean and variance of the time series data do not change over time [37]. The ARIMA model is a combination of two models: the Autoregressive model (AR) and the Moving Average model (MA) connected by differencing (Integration) [38]. The order of the ARIMA model is usually denoted by ARIMA (*p*, *d*, *q*) where *p* denotes the degree to which the current state depends on its past values, *q* represents the order of the moving average process, which accounts for the influence of previous forecast errors while *d* indicates the order of non-seasonal differencing needed to achieve stationarity while integrating both AR and MA parts [14]. The ARIMA model is expressed as;

1 $$\begin{aligned} y_t' = c + \varphi _1y_{t-1}'+\cdots +\varphi _py_{t-p}'+ \theta _1\epsilon _{t-1}+\cdots +\theta _p\epsilon _{t-q}+\epsilon _t, \end{aligned}$$

where $y_t'$ denotes the differenced series, *c* is a constant, $\epsilon _t$ is a white noise term, $\epsilon _t \sim N(0,\sigma ^2)$, $\varphi$’s and $\theta$’s are the coefficients of the AR and MA parts respectively. The autocorrelation function (ACF) and partial autocorrelation function (PACF) are helpful in identifying the parameters for an ARIMA model, where ACF helps determine the moving average order *q* while PACF aids in determining the autoregressive order *p* [37, 39].

**Fig. 2:**
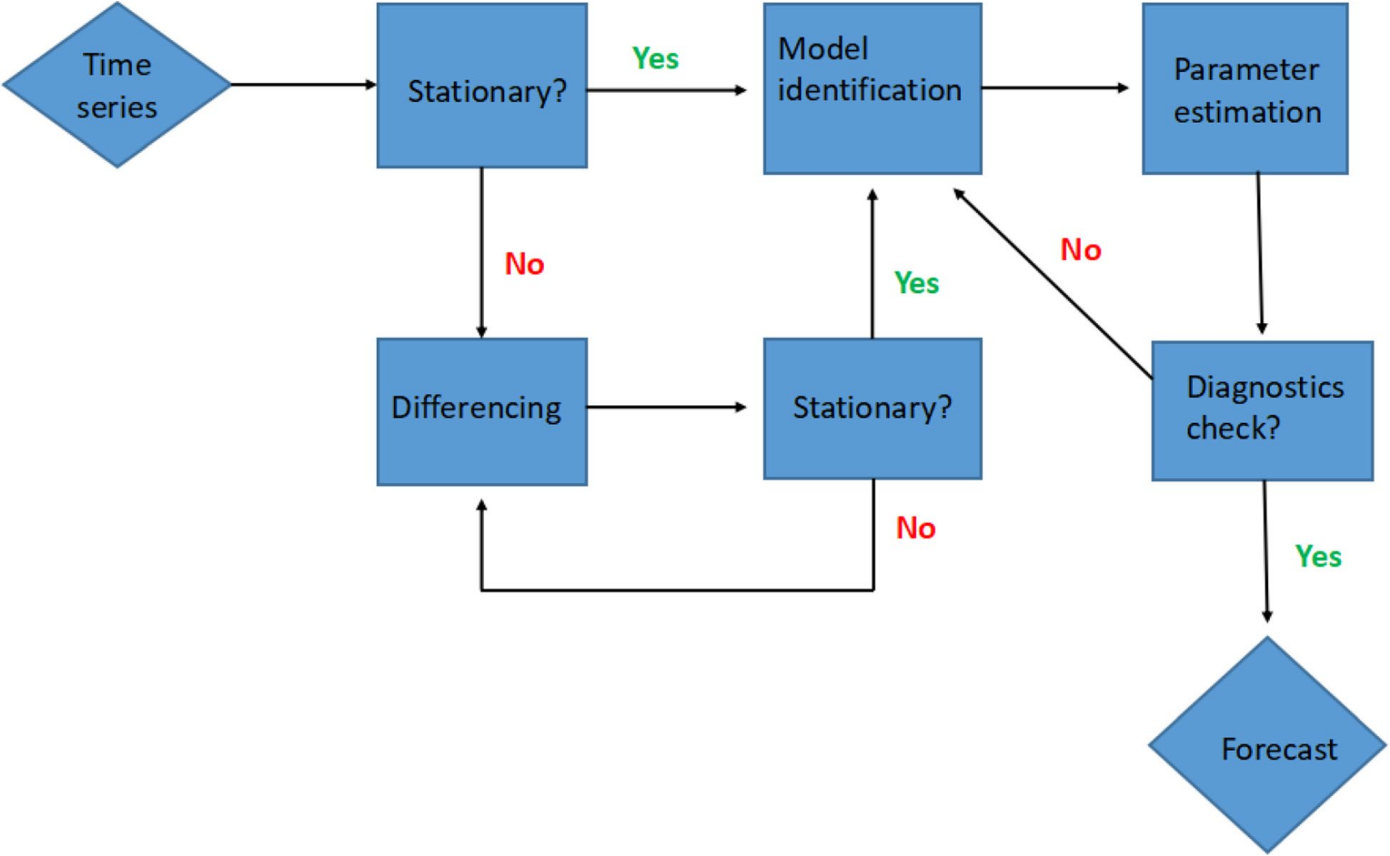
Flow chart of ARIMA forecasting process

The ARIMA modelling procedure firstly begins with a stationarity test, usually performed using the Augmented Dickey-Fuller (ADF) test [39, 40]. Transformations like logarithmic scaling or differencing are used to make a non-stationary time series stationary [40]. The order of differencing *d* is chosen based on the transformations performed during the stationarity check [39, 40]. The next step is model identification, which involves identifying the values of *p* and *q* by visualizing the ACF and PACF plots [37, 39, 40]. Once the model has been specified, estimation of the model parameters $\varphi _i$ and $\theta _j$ will be performed using the maximum likelihood estimate or other parameter estimation methods [37, 40]. Thereafter, the model is validated using statistical tests like the Ljung-Box test to make sure the residuals behave like white noise, which shows that the model has correctly captured the structure of the time series [40]. Once this is done, the Akaike Information Criterion (AIC), Corrected Akaike Information Criterion (AICc), or the Bayesian Information Criterion (BIC) are used in model selection; with the model having the lowest criterion value chosen [39, 40]. Finally, the model can be used to make forecast after it has been validated [40]. The flow chart of the ARIMA forecasting process is shown in Fig. 2.

The parameters (*p*, *d*, *q*) of an ARIMA model can be determined automatically by leveraging statistical methods and computational capabilities using the auto.arima() function in R [37]. By optimizing information criteria like the AIC or BIC, auto.arima automatically chooses the best ARIMA model [40]. A standard procedure when selecting models is to divide the available data into training and test data. The training data is used to estimate any forecasting method parameters, and the test data is used to evaluate the accuracy of the forecast [39, 41].

### Wavelet analysis

While Fourier analysis can effectively measure the constant periodic components of a time series, it cannot describe signals whose frequency content varies over time [23, 24]. Thus, a Fourier decomposition can identify all the frequency components present in a signal but does not indicate when these components occur [24, 42]. Wavelets analysis circumvents the limitation of Fourier analysis by providing effective localization in both time (via translations) and frequency (via dilations), making it well-suited for investigating the temporal evolution of aperiodic and transient signals [42]. Wavelet analysis allows us to examine multi-scale, non-stationary time-series data and uncover hidden features that would otherwise remain unnoticed [24, 25, 43].

**Fig. 3:**
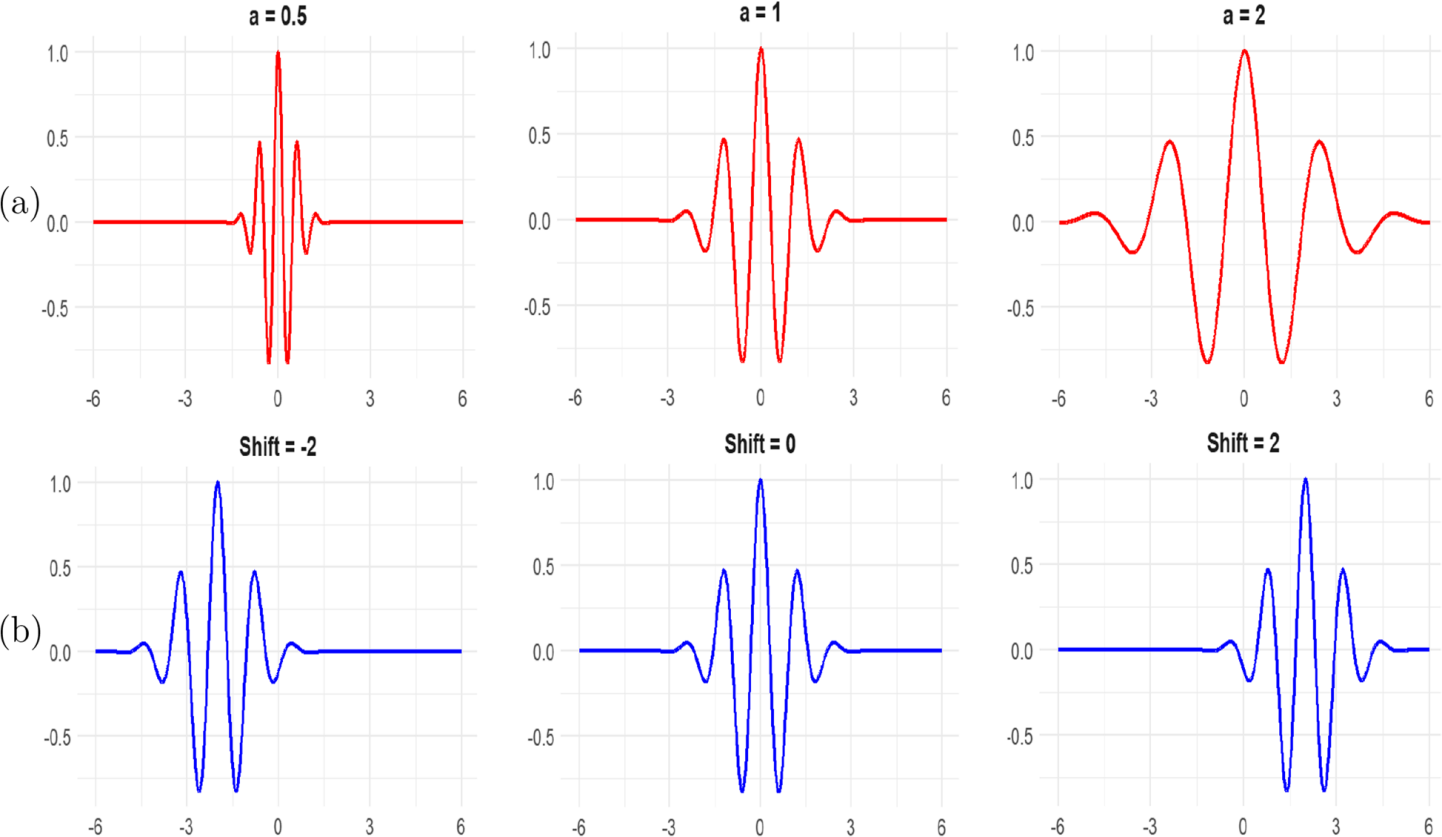
Morlet wavelets at different: **a** scales and **b** translations (shifts)

The continuous wavelet transform of a time series *x*(*t*) with respect to a mother wavelet $\psi (t)$ is given by [25]

2 $$\begin{aligned} W_x(a,s)=\frac{1}{\sqrt{a}}\int _{-\infty }^{\infty } x(t) \psi ^*\left( \frac{t-s}{a}\right) dt, \end{aligned}$$

where * denotes the complex conjugate form, the parameter *a* represents the dilation (scale factor) while *s* represents the translation (time shift). Thus, the wavelet coefficients $W_x(a,s)$ denote the contribution of the scales *a* at various time positions *s*. The factor $\frac{1}{\sqrt{a}}$ helps to normalize the wavelets, ensuring they have a uniform variance of 1 across all scales *a*, thereby making them comparable. The wavelet transform (2) can therefore be viewed as a cross-correlation of a time-series *x*(*t*) with a family of wavelets $\psi \left( \frac{t-s}{a}\right)$ of different scales *a*, at different time positions *s*. Several wavelet functions are used in time series analysis with the Morlet wavelet and the Mexican hat being the two most common continuous wavelets [42]. Figure 3 shows the Morlet wavelets at different scales and translation. The Morlet wavelet detects high-frequency components, oscillates rapidly, and is compressed at low scales. At high scales, it is stretched, oscillates slowly, and detects low-frequency components.

## Results and discussions

**Fig. 4:**
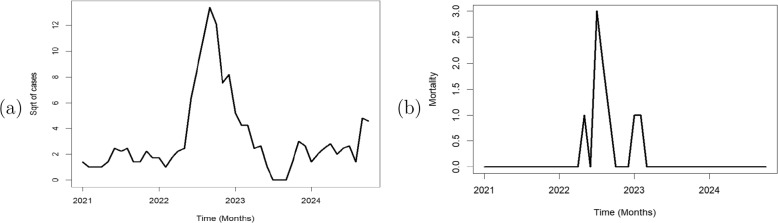
Time series plot of **a** normalised mpox cases. **b** mpox mortality in Nigeria

A square root transformation was performed to reduce the variance of the mpox cases, normalise it and improve it’s stationarity. The normalised times series plot of mpox cases and the time series plot of mpox mortality are shown in Fig. 4. The mpox cases plot depicts the time series of data points over several months, spanning from January 2021 to October 2024 for the mpox cases in Nigeria. The y-axis (labeled “Sqrt of cases”) shows a variable with the square root of values between 0 and above 100 cases, while the x-axis shows time in months. The plot includes a prominent spike around mid-2023, followed by a decline, with some fluctuations near the end. The time series plot suggests a periodic or seasonal process with a large anomaly or peak in mid-2023. It indicates a one-time event causing a sharp increase or an epidemic of mpox in Nigeria followed by a return to baseline.

**Fig. 5:**
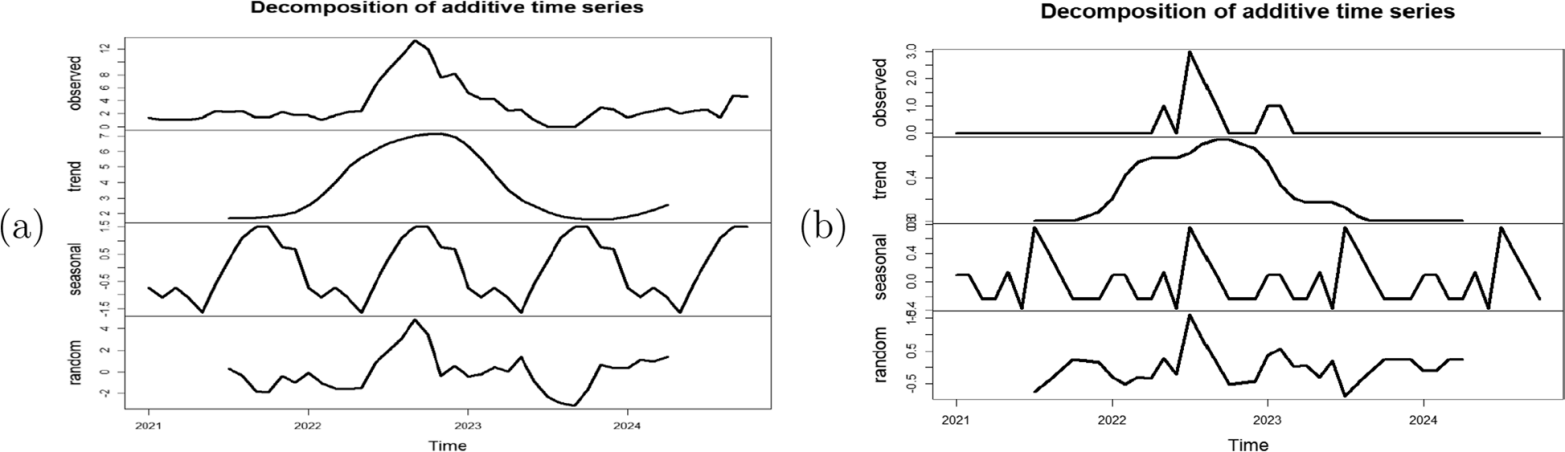
A decomposition of (**a**) mpox cases and (**b**) mortality time series in Nigeria

The decomposition of the normalised mpox cases and mortality time series data is shown in Fig. 5. The additive time series decomposition shows the trend, seasonal and remainder components of the data. Both the cases and mortality data were divided into two; the training set (70%) and the test set (30%). The auto.arima() function was applied on the training sets to generate plausible models. Seven ARIMA models were generated for the mpox cases - ARIMA(4,0,0), ARIMA(4,0,1), ARIMA(5,0,1), ARIMA(5,0,0), ARIMA(3,0,0), ARIMA(2,0,2) and ARIMA(2,0,0) with their respective AIC values given as 121.38, 121.34, 122.39, 120.53, 122.07, 127.76 and 122.95. similarly, seven candidate ARIMA models were identified for the mpox mortality training set, along with their corresponding AIC values: ARIMA(1,0,0) - 64.36, ARIMA(2,0,0) - 66.05, ARIMA(0,0,1) - 66.45, ARIMA(1,0,1) - 66.16, ARIMA(1,0,2) - 67.30, ARIMA(2,0,2) - 67.43, and ARIMA(2,0,1) - 67.47.

**Fig. 6:**
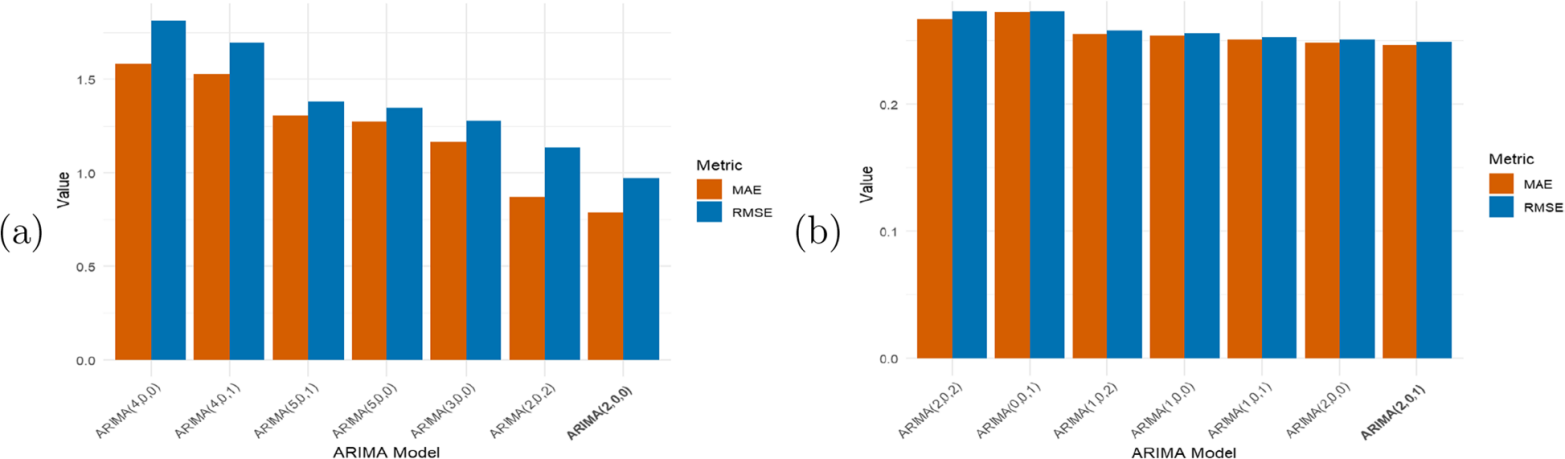
Comparison measures of ARIMA models for (**a**) mpox cases and (**b**) mortality time series in Nigeria

The precision and predictive quality of each of the two sets of seven ARIMA models was evaluated using the corresponding test data. The evaluation metrics used are the root mean square error (RMSE) and the mean absolute error (MAE). The values of these evaluation metrics are presented in Table 1. A visual representation of the comparison of RMSE and MAE for the two sets of ARIMA models is shown in Fig. 6. For the mpox cases, ARIMA(2,0,0) has the lowest RMSE (0.9722126) and MAE (0.7869304) values, and thus offered the best fit for the cases data. ARIMA(2,0,1) has the lowest RMSE (0.2488994) and MAE (0.2463825) values, and provided the best fit for the mpox mortality data.

**Table 1:** Evaluation of predictive performance across selected ARIMA models for (a) mpox cases and (b) mpox mortality

(a) Mpox cases			(b) Mpox mortality		
Model	RMSE	MAE	Model	RMSE	MAE
ARIMA(4,0,0)	1.8156085	1.5849722	ARIMA(2,0,2)	0.2732354	0.2665275
ARIMA(4,0,1)	1.6957971	1.5283227	ARIMA(0,0,1)	0.2727688	0.2722160
ARIMA(5,0,1)	1.3787837	1.3053530	ARIMA(1,0,2)	0.2576634	0.2553258
ARIMA(5,0,0)	1.3487984	1.2766706	ARIMA(1,0,0)	0.2556817	0.2537690
ARIMA(3,0,0)	1.2784915	1.1680685	ARIMA(1,0,1)	0.2527727	0.2506824
ARIMA(2,0,2)	1.1355074	0.8708468	ARIMA(2,0,0)	0.2505548	0.2483190
**ARIMA(2,0,0)**	**0.9722126**	**0.7869304**	**ARIMA(2,0,1)**	**0.2488994**	**0.2463825**

**Fig. 7:**
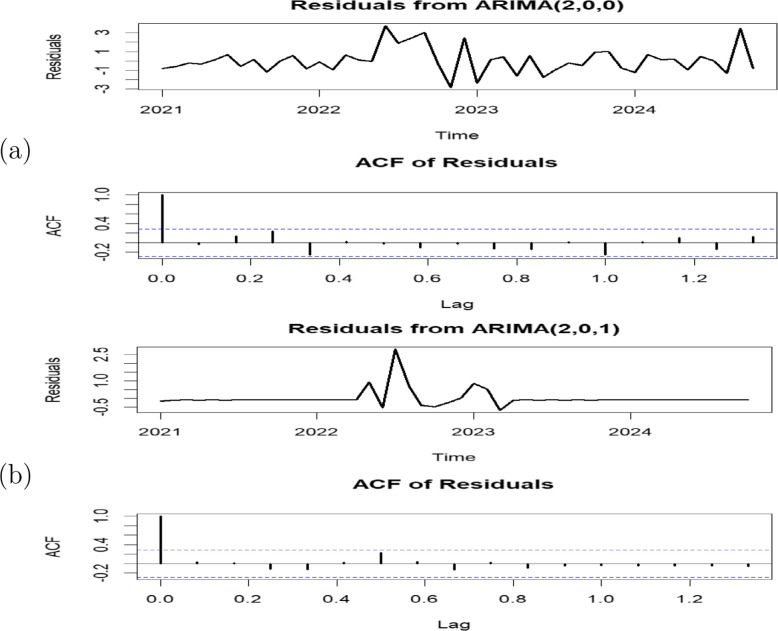
Plots of (**a**) ARIMA(2,0,0) residuals and (**b**) ARIMA(2,0,1) residuals. The blue dashed lines indicate the significance threshold

Diagnostic checks were carried out on the residuals of ARIMA(2,0,0) and ARIMA(2,0,1) to determine whether the estimated models were able to extract adequate information from the data. The Ljung-Box test was used to test for autocorrelation remaining in the residuals. The *p*-values for the Ljung-Box Q-statistics are much higher than 0.05, suggesting nonsignificance. Figure 7 shows the plots of the residuals of the two models. The ACF plots of the residuals for both ARIMA(2,0,0) and ARIMA(2,0,1) show that, except for the first lag, there are no significant autocorrelations.

**Fig. 8:**
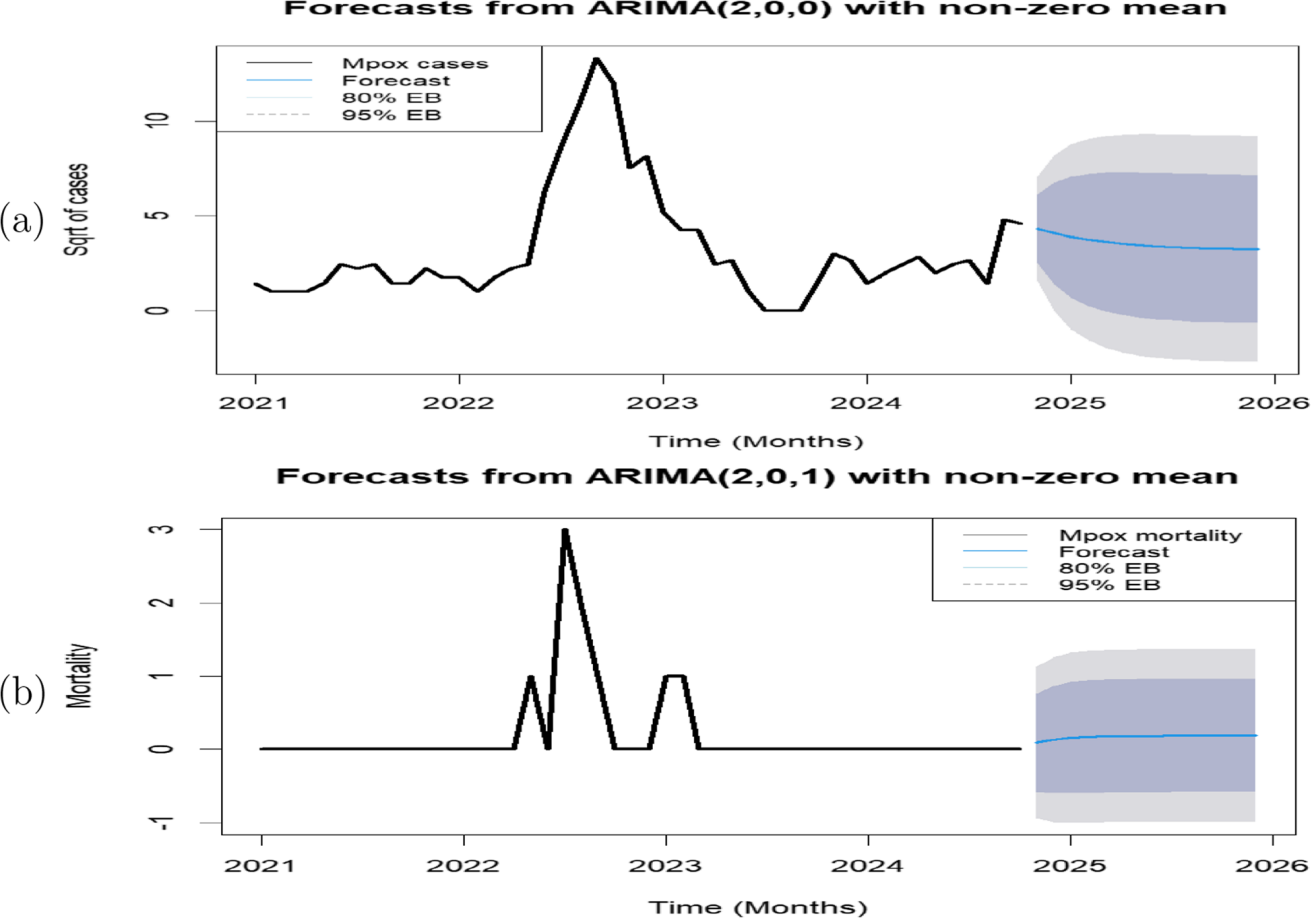
ARIMA model forecast for (**a**) mpox cases and (**b**) mortality in Nigeria from November 2024 to December 2025. The black line represents observed data, while the blue line indicates forecasts with 80% and 95% confidence intervals shown in shaded regions

The selected ARIMA models were used to forecast the mpox cases and mortality for 14 months beginning from November 2024 to December 2025. The plots of the forecasted mpox cases and mortality are depicted in Fig. 8. The confidence intervals, which show the range in which the actual forecasted cases and mortality values are expected to fall, are represented by the shaded areas surrounding the forecast lines. The area shaded grey represents a 95% confidence interval (CI) while the darker shaded area represents an 80% confidence interval (CI). The forecast results indicate a gradual downward trend in mpox cases in Nigeria starting from November 2024 to December 2025. The forecast values of mpox cases for 14 months are presented in Table 2.

**Table 2:** Forecasted values of Mpox cases in Nigeria from November 2024 to December 2025

Time	Forecast	Lower 80% CI	Upper 80% CI	Lower 95% CI	Upper 95% CI
Nov 2024	**18.68**	6.32	37.57	2.42	50.22
Dec 2024	**16.72**	1.96	45.94	0.00	67.26
Jan 2025	**15.19**	0.49	50.38	0.99	77.30
Feb 2025	**14.04**	0.06	52.45	2.56	82.68
Mar 2025	**13.16**	0.00	53.23	3.93	85.33
Apr 2025	**12.51**	0.05	53.36	4.96	86.49
May 2025	**12.01**	0.13	53.19	5.71	86.86
Jun 2025	**11.63**	0.21	52.91	6.25	86.84
Jul 2025	**11.34**	0.27	52.59	6.63	86.65
Aug 2025	**11.11**	0.32	52.29	6.90	86.39
Sep 2025	**10.94**	0.36	52.03	7.10	86.13
Oct 2025	**10.81**	0.39	51.81	7.24	85.89
Nov 2025	**10.71**	0.41	51.63	7.35	85.68
Dec 2025	**10.63**	0.43	51.48	7.43	85.51

### Wavelet power spectrum

**Fig. 9:**
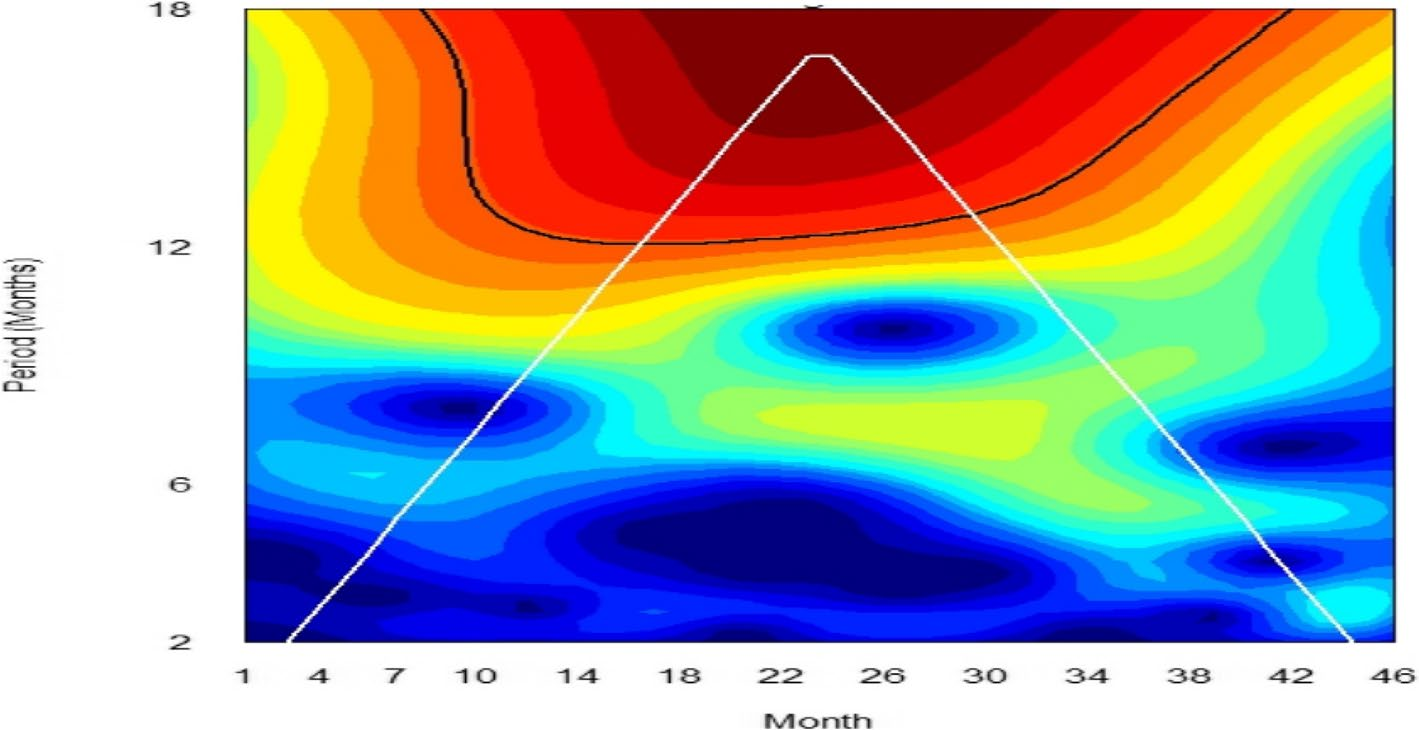
Wavelet Spectrum of mpox in Nigeria

**Fig. 10:**
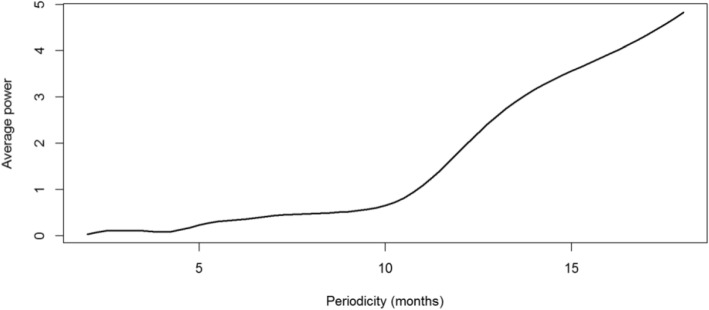
Plot of average power spectrum of mpox in Nigeria

**Fig. 11:**
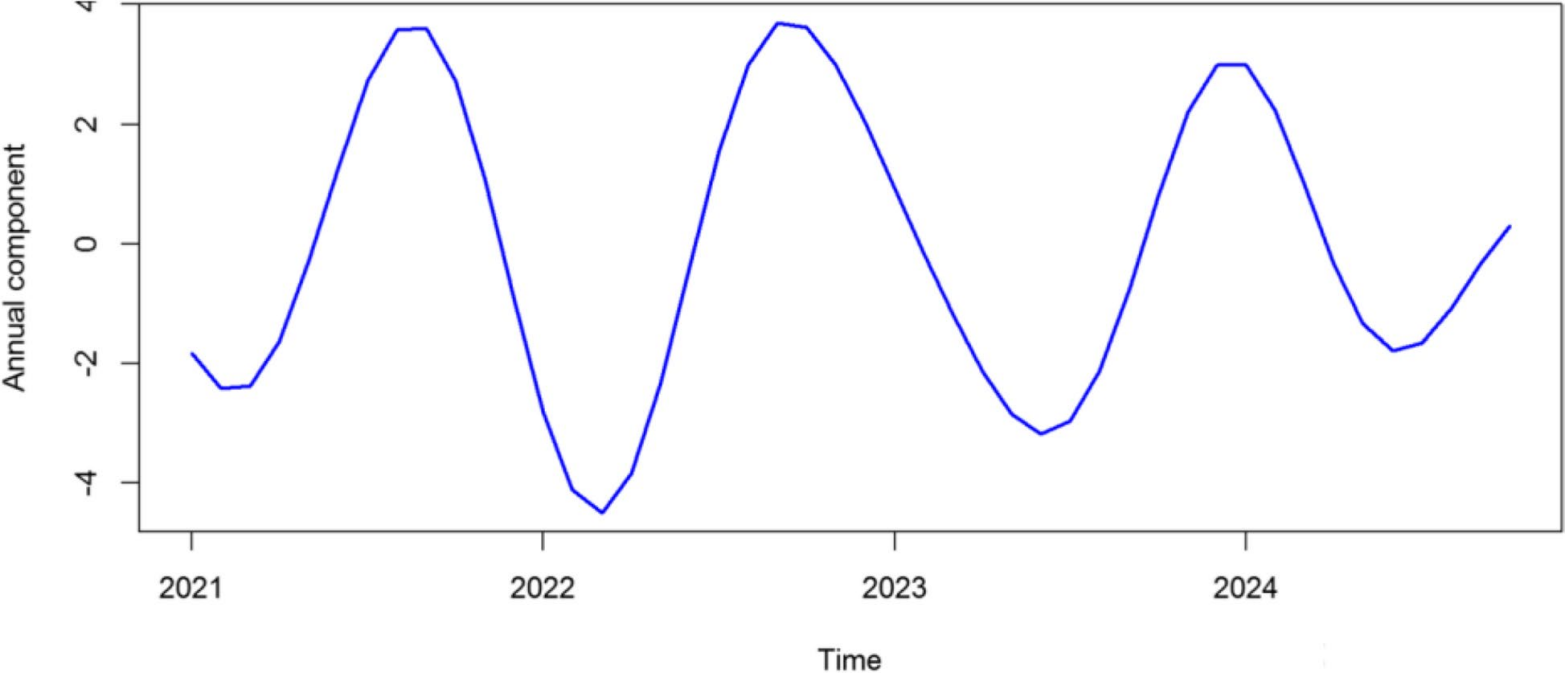
Plot of annual cycles of mpox in Nigeria

**Fig. 12:**
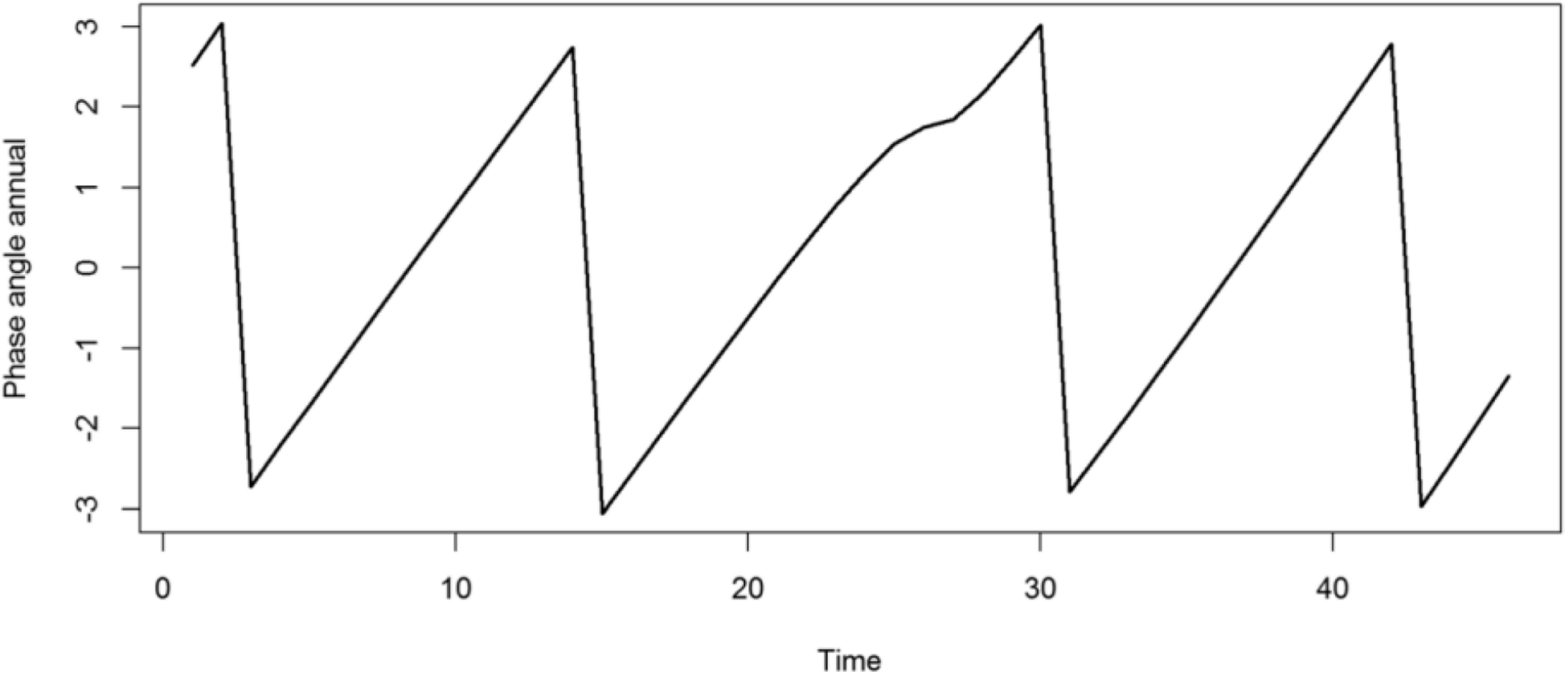
Plot of phase angle annual

Figure 9 presents the local wavelet power spectrum of mpox incidence in Nigeria between January 2021 and October 2024. At such time scales, the deep blue regions represent low power, indicating weak periodicity, while the deep red regions indicate high power, suggesting strong periodicity. The deep red area between 12–18 periodic band indicates a strong annual cycle in the incidence of mpox in Nigeria while the blue and green area around the 2–6 periodic band suggests presence of secondary fluctuations or short-term outbreak patterns. The yearly cycle at the 12–18 periodic band is at its strongest between the time period June 2022 and June 2023 indicating that mpox outbreak became more frequent during this period. The cone of influence (COI), which indicates the region where edge effects are significant, is described with a white triangular line. Figure 10 shows the average wavelet power spectrum of mpox cases in Nigeria during the period under consideration. Low power is observed between the 2–6 months periodic band indicating weak periodicity and short-term fluctuations. Power begins to increase at the 6 month periodic band and shows a significant increase in the 12–18 periodic band for the time period June 2022 to June 2023, confirming the presence of a strong yearly cycle. Figure 11 presents the plot of the annual cycles of mpox in Nigeria. The plot of the phase angle is shown in Fig. 12.

## Conclusion

The study highlights the trends and hidden patterns of mpox outbreaks in Nigeria using the ARIMA forecasting model and wavelet analysis. The forecast suggests a decline in the incidence of mpox in Nigeria. It indicates a total expected mpox cases of about 180 for the next 14 months with zero mortality. Wavelet analysis was further used to decompose the data into different frequency componenets and reveal hidden patterns. Unlike the ARIMA model which assumes stationarity and is not suitable for non-stationary time series, wavelet analysis is capable of handling non-stationarity, a common property of epidemiological time series data. The wavelet power spectrum shows a noticeably strong annual cycle between June 2022 and June 2023. The study offers useful insights into future mpox trends in Nigeria and helps policymakers make informed decisions on how to adequately plan for continuous surveillance, resource allocation, and targeted intervention strategies to mitigate potential outbreaks. To sustain the forecasted downward trend in mpox cases in the coming months, it is pertinent that the National Mpox Technical Working Group (TWG) of NCDC continue to scale up vaccination coverage and improve surveillance especially in high risk areas. However, the study is not without some limitations. Firstly, the historical dataset used is quite limited and can only give preliminary insights into short-term trends of mpox cases in Nigeria. In order to guide interpretation for the purpose of decision making, 80% CI and 90% CI were used to quantify the forecast uncertainty. Future access to larger datasets will our improve model’s accuracy and help forecasts’ reliability in policymaking. Another significant drawback of the ARIMA model used in the study is that it only takes into account historical data, and ignores other external variables such as the coverage rates of interventions, changes in policy, or behavioural shifts that could have an impact on future patterns [40]. To address this limitation, future study intends to apply the AutoRegressive Integrated Moving Average with Exogenous Variables (ARIMAX) model using vaccination data from the recently launched mpox vaccination campaign in Nigeria. Furthermore, ARIMA models are limited by their assumption of linearity, which prevents them from capturing nonlinear behaviour common with most epidemiological data [44]. As such, future studies will employ nonlinear models such as neural-network based teachniques to improve forecast accuracy.

## Data Availability

All data used is on the Nigeria Centre for Disease Control and Prevention (NCDC) dashboard.

## Abbreviations

WHO: World Health Organization
PHEIC: Public Health Emergency of International Concern
ARIMA: Auto-Regressive Integrated Moving Average
ARIMAX: Auto-Regressive Integrated Moving Average with Exogenous Variables
MPXV: Mpox Virus
DRC: Democratic Republic of Congo
NCDC: Nigeria Centre for Disease Control and Prevention
TWG: Technical Working Group
FCT: Federal Capital Territory
AR: Auto-Regressive
MA: Moving Average
ACF: Autocorrelation Function
PACF: Partial Autocorrelation Function
ADF: Augmented Dickey-Fuller
AIC: Akaike Information Criterion
AICc: Corrected Akaike Information Criterion
BIC: Bayesian Information Criterion
RMSE: Root Mean Square Error
MAE: Mean Absolute Error
COI: Cone of Influence
CI: Confidence Interval
